# Transcriptome analysis of gravitational effects on mouse skeletal muscles under microgravity and artificial 1 *g* onboard environment

**DOI:** 10.1038/s41598-021-88392-4

**Published:** 2021-04-28

**Authors:** Risa Okada, Shin-ichiro Fujita, Riku Suzuki, Takuto Hayashi, Hirona Tsubouchi, Chihiro Kato, Shunya Sadaki, Maho Kanai, Sayaka Fuseya, Yuri Inoue, Hyojung Jeon, Michito Hamada, Akihiro Kuno, Akiko Ishii, Akira Tamaoka, Jun Tanihata, Naoki Ito, Dai Shiba, Masaki Shirakawa, Masafumi Muratani, Takashi Kudo, Satoru Takahashi

**Affiliations:** 1 Mouse Epigenetics Project, ISS/Kibo Experiment, Japan Aerospace Exploration Agency (JAXA), Ibaraki, 305-8505 Japan; 2 JEM Utilization Center, Human Spaceflight Technology Directorate, JAXA, Ibaraki, 305-8505 Japan; 3 Doctoral Program in Biomedical Sciences, Graduate School of Comprehensive Human Sciences, University of Tsukuba, Ibaraki, 305-8575 Japan; 4 Department of Genome Biology, Faculty of Medicine, University of Tsukuba, Ibaraki, 305-8575 Japan; 5 Laboratory Animal Resource Center in Transborder Medical Research Center, Department of Anatomy and Embryology, Faculty of Medicine, University of Tsukuba, Ibaraki, 305-8575 Japan; 6 Ph.D. Program in Human Biology, School of Integrative and Global Majors, University of Tsukuba, Ibaraki, 305-8575 Japan; 7 Master’s Program in Medical Sciences, Graduate School of Comprehensive Human Sciences, University of Tsukuba, Ibaraki, 305-8575 Japan; 8 Department of Neurology, Faculty of Medicine, University of Tsukuba, Ibaraki, 305-8575 Japan; 9 Department of Cell Physiology, The Jikei University School of Medicine, Tokyo, 105-8461 Japan; 10 Laboratory of Molecular Life Science, Institute of Biomedical Research and Innovation, Foundation for Biomedical Research and Innovation at Kobe (FBRI), Kobe, 650-0047 Japan

**Keywords:** Musculoskeletal system, Transcriptomics, Functional clustering, Gene ontology, Gene regulatory networks, Gene expression, RNA sequencing

## Abstract

Spaceflight causes a decrease in skeletal muscle mass and strength. We set two murine experimental groups in orbit for 35 days aboard the International Space Station, under artificial earth-gravity (artificial 1 *g*; AG) and microgravity (μ*g*; MG), to investigate whether artificial 1 *g* exposure prevents muscle atrophy at the molecular level. Our main findings indicated that AG onboard environment prevented changes under microgravity in soleus muscle not only in muscle mass and fiber type composition but also in the alteration of gene expression profiles. In particular, transcriptome analysis suggested that AG condition could prevent the alterations of some atrophy-related genes. We further screened novel candidate genes to reveal the muscle atrophy mechanism from these gene expression profiles. We suggest the potential role of *Cacng1* in the atrophy of myotubes using in vitro and in vivo gene transductions. This critical project may accelerate the elucidation of muscle atrophy mechanisms.

## Introduction

Gravity is the most constant factor affecting the entire process of evolution of organisms on Earth. As adapting to a changing environment is key for any organism’s survival, the constant mechanical stimulus of gravitational force has been shared by all organisms on Earth through evolution^1^. To elucidate how mammals respond to gravity, space experiments with mice are quintessential; however, they require specialized habitats applicable to the space environment.

Skeletal muscle is one of the most robustly plastic tissues that can adapt its structure and metabolism in response to various conditions^2,3^. Under disuse conditions such as prolonged time in bed, skeletal muscles undergo atrophy due to decreased protein synthesis and increased protein degradation^4^. Spaceflight is well-known to induce muscle atrophy and weakness in rats^5,6^ and mice^7^. In humans^8–10^, muscle atrophy during spaceflight can cause severe medical problems to astronauts upon return to Earth. Previous studies have shown that muscle atrophy and partial shift of muscle fibers from oxidative toward a more glycolytic phenotype were induced in space-flown rats and mice, as well as humans^11^. Therefore, there is a critical need for a deeper understanding of the molecular mechanisms responsible for muscle atrophy under microgravity and partial gravity (less than 1 *g*).

The effect of spaceflight on skeletal muscles has been examined in rats and mice, comparing a spaceflight group with a ground control group^6,12,13^. However, such studies did not consider various conditions, including space radiation, the microbial environment, lack of convection, and shock during the launch and return phases that differ between spaceflight and conventional control experiments. Comparing between the two control groups was needed to rule out the confounding factors originating from different housing conditions, such as the effect of short-term gravitational changes during launch and landing in spaceflight. Therefore, we developed the multiple artificial-gravity research system (MARS) and succeeded in housing mice under microgravity and an artificial 1 *g* onboard environment to study the effect of gravity on mammals^14–16^. In the first Mouse Habitat Unit-1 (MHU-1) mission, 12 mice were housed in the International Space Station (ISS) using MARS under either microgravity or artificial 1 *g* for 35 days in 2016. Each male mouse was reared in an individual cage to avoid unexpected fighting between mice, and all mice returned to the Earth alive. We set an artificial 1 *g* onboard environment induced by centrifuging cages as the novel control. This is because artificial 1 *g* onboard was a novel experimental group, we employed a comprehensive assessment against the effects of microgravity and artificial 1 *g*. Our findings from the MHU-1 mission were as follows; retinal apoptosis caused by spaceflight was prevented in artificial 1 *g*^17^; downregulation of transcription factors GATA1 and Tal1 (regulator of erythropoiesis) in the immune system was observed in microgravity^18^; and the physiological functions of the male reproductive organs, such as sperm function and offspring viability, were maintained in microgravity^19^.

In this study, we aimed to elucidate the effect of an artificial 1 *g* onboard environment on skeletal muscles at the molecular level. We investigated the histological characterization of muscle fibers and gene expression from the JAXA mission (MHU-1). We found that an artificial 1 *g* onboard environment prevented not only decreased muscle mass and change in fiber type composition but also the alteration of gene expression profiles in soleus muscle during spaceflight. Transcriptome analysis also suggested that AG condition could prevent the alterations of well-known atrophy-related genes. We further screened the candidate genes associated with muscle atrophy using these gene profiles. We identified *Cacng1* that could play a functional role in myotubes atrophy by in vitro and in vivo gene transductions. Therefore, our project demonstrates the utility of spaceflight datasets using an artificial 1 *g* onboard environment for elucidating the basis of muscular adaptation during spaceflight.

## Results and discussion

### An artificial 1 *g* onboard environment prevents changes in muscle mass and the fiber type composition of the soleus muscle under microgravity

**Figure 1:**
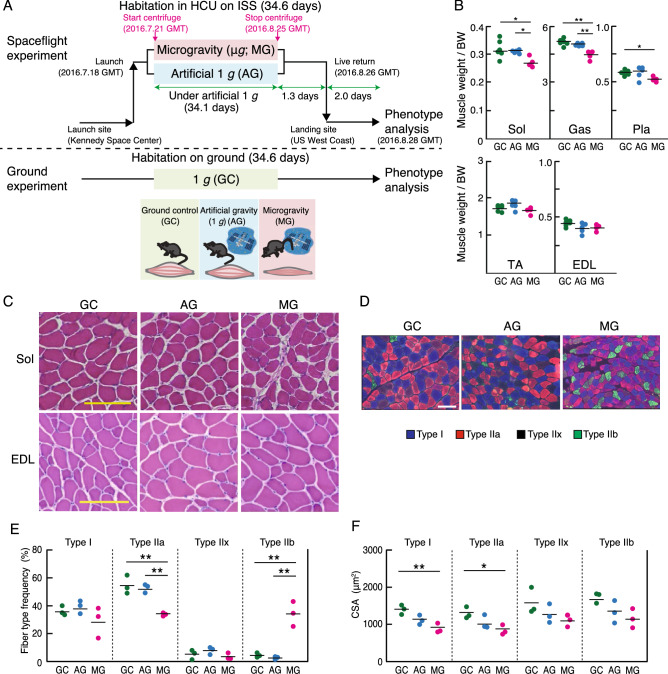
Characterization of the hindlimb muscles among three experimental groups: GC, AG, and MG. (**A**) Overview of the MHU-1 study. Twelve male mice were launched by SpaceX-9 from the Kennedy Space Center in Florida on July 18, 2016. After arrival at the ISS, six of the mice were housed in HCU under microgravity (MG) for approximately 34.6 days, while the other six mice were housed in an artificial 1 *g* onboard environment (AG) induced by centrifugation for approximately 34.1 days during the approximate 37-day berthing period. After approximately 35 days of habitation on the ISS, all 12 mice were loaded into the Dragon capsule and kept under microgravity for approximately 1.3 days before undocking. The capsule subsequently splashed down in the Pacific Ocean near Southern California on August 26, 2016. The mice were then transported to a laboratory for behavioral tests and other analyses after approximately 2 days of undocking. Graphical representations of the study design using the multiple artificial-gravity research system (MARS). Three experimental groups, namely, ground control (GC), artificial 1 *g* onboard environment (AG), and microgravity (MG) in the ISS, are color-coded as green, blue, and red, respectively, in the following bar graphs. (**B**) Quantification of the change in individual skeletal muscle weights: soleus (Sol), gastrocnemius (Gas), plantaris (Pla), tibialis anterior (TA), and extensor digitorum longus (EDL), among GC (n = 6), AG (n = 5), and MG (n = 4) groups, normalized by body weight and graphed in the Tukey boxplot. *P*-value from Tukey’s test is indicated as follows: *P < 0.05 and **P < 0.01. (**C**) Hematoxylin–eosin staining of soleus and EDL sections. Scale bars indicate 100 μm. (**D**) Immunostaining of the myosin heavy chain using muscle fiber-specific antibodies (type I; blue, type IIa; red, type IIb; green) in the soleus muscle. The unstained fibers were predicted to be of type IIx fibers (black). Scale bars indicate 100 μm. (**E**, **F**) Frequency (**E**) and cross-sectional areas (CSA) (**F**) of each fiber type in the soleus muscles of the GC (n = 3), AG (n = 3), and MG (n = 3). *P*-value from Student’s *t*-test is indicated as follows: *P < 0.05 and **P < 0.01.

Twelve mice were housed onboard the ISS for 35 days. Half of them were housed under microgravity (MG), whereas the other 6 mice were housed under an artificial 1 *g* onboard environment (AG) induced by centrifuging (Fig. 1A). Since previous studies using ground control group did not distinguish the effect of microgravity from other various conditions such as space radiation and lack of convection, we examined whether artificial 1 *g* exposure prevents the reduction in muscle mass induced by spaceflight in mice. To compare the effects of microgravity and an artificial 1 *g* onboard environment on skeletal muscle, we measured the wet muscle mass of hindlimb skeletal muscles, namely soleus, gastrocnemius, plantaris, tibialis anterior (TA), and extensor digitorum longus (EDL) (Fig. 1B). The muscle weights were normalized using the body weight of each mouse. Previously, we reported that AG completely inhibited the effect of microgravity on soleus, which is the main postural muscle of hindlimb and gastrocnemius muscles, compared with MG and ground control (GC), which was housed on the ground for the same duration using similar cages and processed with the same method as spaceflight mice^14^. The weight of the plantaris muscle in MG was also significantly decreased compared with that of GC, although the weight of the plantaris muscle in MG tended to decrease compared with AG. The weight of the TA and EDL muscles showed no significant differences among the three groups. Hematoxylin–eosin (HE) staining of freshly-embedded soleus (predominantly slow-twitch fibers) and EDL (predominantly fast-twitch fibers) muscle sections, which are composed of different types of muscle fibers, showed the absence of major pathological anomalies, such as central nuclei, immune cell infiltration, and myofiber degeneration in all analyzed muscles from each experimental group: GC, AG, and MG (Fig. 1C). The previous studies have shown that the change in muscle fiber type composition in both type I and type II is observed in the soleus muscle after spaceflight in mice, rats, and humans^11,20^. Additionally, atrophy is reportedly more pronounced in type I than in type II fibers of the soleus muscle. Therefore, immunofluorescence staining of the different myosin heavy chain (MyHC) isoforms (i.e., types I, IIa, IIb) was performed for evaluating whether microgravity or artificial 1 *g* exposure in space affected muscle fiber type composition in the soleus muscle (Fig. 1D). The type IIx fibers were identified by the absence of immunoreaction during triple immunostaining. We quantified the fiber type frequency and cross-sectional area (CSA) of the myofibers by immunofluorescence staining of the soleus muscle (Fig. 1E, F). The ratio of type IIa fibers was reduced, while that of type IIb fibers increased in the soleus muscle under MG compared to that of under GC. The decrease of type IIa fibers ratio and the increase of type IIb fibers ratio were completely prevented in AG, implying that the change in fiber type composition induced by spaceflight was mainly caused by unloading due to microgravity. Additionally, the CSA significantly decreased in type I and type IIa fibers in MG compared with those in GC. These reductions in fibers were not significant when comparing GC and AG (Fig. 1F), suggesting that AG tends to prevent the reduction of CSA during spaceflight. These inflight 1 *g* control results suggested that an artificial 1 *g* onboard environment using centrifuge could prevent change in the fiber type composition and muscle atrophy during spaceflight in both the fast- and slow-twitch fibers of the soleus muscle. In contrast, no changes in the CSAs of EDL muscles were observed among the three groups (Supplementary Fig. 1). These results demonstrated that the postural muscles of lower limbs were highly responsive to microgravity. Our histological data supported previous findings of fiber composition change exclusively observed in the soleus muscle exposed to microgravity for 30 days using BION-M1^13^. In addition, change in fiber type composition was observed in the soleus muscle, but not the EDL muscle, of mice housed onboard for as long as 91 days (MDS mission, 2009)^12^. Our findings showed that an artificial 1 *g* onboard environment is sufficient to prevent change in the fiber type composition, and has a potential to partially prevent muscle atrophy in the soleus muscle during spaceflight.

### Artificial 1 *g* onboard environment prevents the alteration of gene expression under microgravity

**Figure 2:**
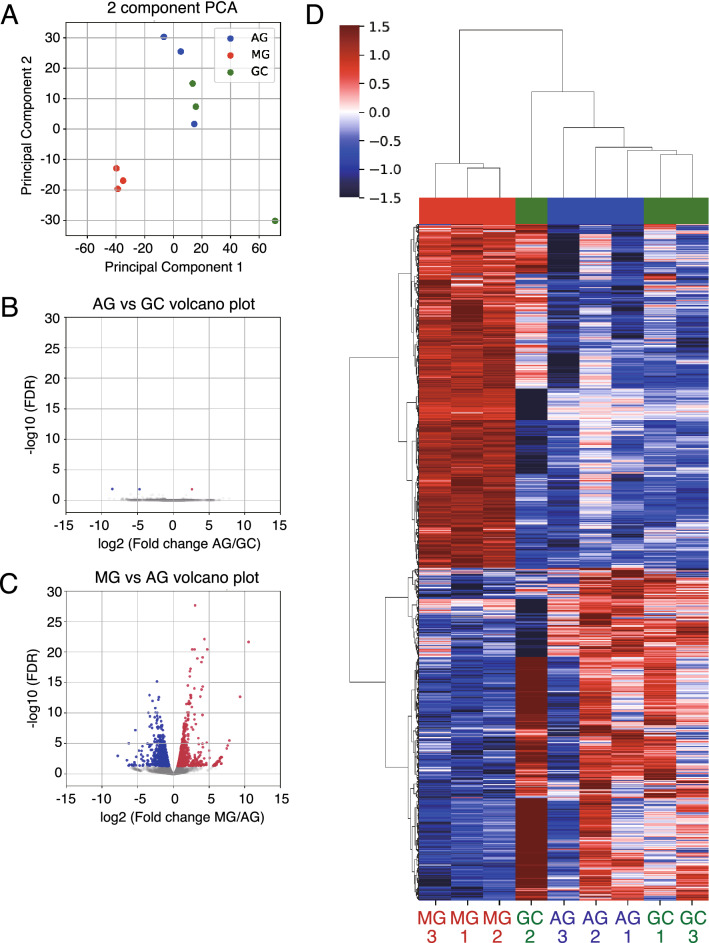
Gene expression profiling in the soleus muscle of GC, AG, and MG. (**A**) PCA of DEGs in at least one comparison among the soleus muscle of GC (n = 3; green), AG (n = 3; blue), and MG (n = 3; red). (**B**, **C**) Volcano plots showing DEGs in the soleus muscle of AG and GC (B), and MG and AG (C). (**D**) Heat map and hierarchical clustering of the gene expression data in the soleus muscle of GC, AG, and MG showing 1,720 DEGs in AG and MG.

The previous studies have compared the global gene expression profile of the soleus muscle following microgravity exposure with that of the ground control group using microarray^13^ and RNA-seq^21^ techniques. However, as inflight 1 *g* exposed control groups have not been used in these studies, it remains to be elucidated whether the loading gravity is sufficient for maintaining the altered gene expression in the soleus muscle during spaceflight. A previous study demonstrated that the largest number of differentially expressed genes (DEGs) is found in the soleus muscle among nine different tissues in mice housed in the ISS for 37 days^22^. Therefore, in order to investigate skeletal muscle adaptation during spaceflight using the MARS platform, we focused on the soleus muscle, which is one of the representative antigravitational muscles. To investigate the comprehensive impact of gene expressions due to microgravity and artificial 1 *g* exposure in spaceflight, we performed transcriptome analysis of the soleus muscles from MG, AG, and GC mice (GC: n = 3; AG: n = 3; MG: n = 3). Principal component analysis (PCA) showed that MG mice’s gene expression profiles considerably differed from those of AG and GC mice (Fig. 2A). In addition, the gene expression profiles between AG and GC are relatively similar with the exception for one GC mouse (Fig. 2A). These results suggested that gravitational change could affect the muscle’s gene expression profile in mice under spaceflight for 35 days rather than other events, such as space radiation. Indeed, comparing the two control groups (i.e., GC and AG), only three genes, namely *Nr4a3*, *Sfrp4*, and *Gm21541,* were differentially regulated in the soleus muscle (Fig. 2B and Supplementary Table 1). The slight difference between these two control groups could be caused by different environmental factors, such as the effect of short-term gravitational changes during launch and landing in spaceflight. Comparing AG and MG, 1,720 genes were differentially regulated in the soleus muscle with 874 upregulated and 846 downregulated genes (Fig. 2C), although 1,992 genes were differentially regulated in the soleus muscle with 810 upregulated genes and 1,182 downregulated genes by comparing GC and MG (Supplementary Fig. 2). These results suggested microgravity may contribute to the change of comprehensive gene expressions. In other words, our results suggested that an artificial 1 *g* onboard environment ensured by centrifuging the cages prevented the alteration of gene expression under microgravity in the soleus muscle. We next focused on the subsequent analysis of genes differentially regulated in MG compared with AG. Hierarchical clustering was performed on DEGs of 1,720 genes (AG vs. MG) with all samples for GC, AG, and MG. The result could also confirm the similarity of gene expression profiles of the two control groups (GC and AG), compared with MG (Fig. 2D).

In summary, we demonstrated that an artificial 1 *g* onboard environment could prevent gene expression changes observed under microgravity, which suggested that gravitational change rather than other events such as space radiation largely affected the gene expression changes in the soleus muscle under spaceflight for 35 days. Besides, the GC experiment using the same cage unit as spaceflight conditions is acceptable for analyzing microgravity’s effect on mice’s muscles in spaceflight.

### An artificial 1 *g* onboard environment could block alteration of atrogene expressions under microgravity

Muscle atrophy is mediated by transcription-dependent regulation of atrogenes (atrophy-related genes), whose expression is coordinately upregulated or downregulated in muscles during systemic wasting states, such as fasting, cancer cachexia, renal failure, and diabetes^23^. In particular, a previous study demonstrated that the E3 ubiquitin ligase MuRF1 (*Trim63*) null (KO) mice showed protection in ground-based models of muscle atrophy under multiple conditions, including denervation and immobilization^24^. However, the deletion of MuRF1 in mice did not prevent muscle atrophy during spaceflight, suggesting that muscle atrophy occurs through different mechanisms during spaceflight compared with those observed in ground-based models of atrophy^21^.

**Figure 3:**
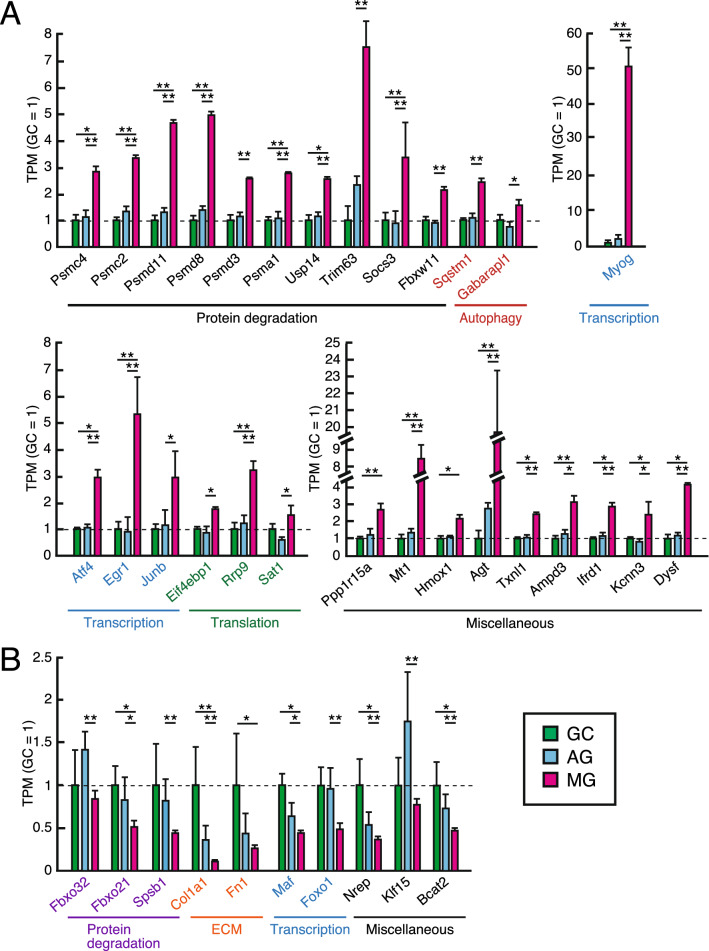
Differentially expressed genes of atrogenes in the soleus muscle of GC, AG, and MG. Bar diagrams showing the gene expression of atrogenes (atrophy-related genes) as significantly upregulated (**A**) and downregulated (**B**) in MG compared with GC and/or AG. FDR-corrected *P*-value from statistical test is indicated as follows: *FDR-corrected *P* < 0.05 and **FDR-corrected *P* < 0.01. GC (Green), AG (Blue), and MG (Red).

Hence, to elucidate the effects of microgravity and an artificial 1 *g* onboard environment on the gene expression profile of atrogenes, we identified genes under the regulation of microgravity by cross-referencing the list of atrogenes^25–28^. RNAseq analysis confirmed the differentially regulated atrogenes in MG compared with GC and/or AG (Fig. 3A, B). Consistent with muscle morphology, gene expressions involved in protein degradation mainly by the ubiquitin–proteasome system were significantly upregulated in MG (Fig. 3A). This finding includes the genes encoding for various subunits of the 26S proteasome, such as subunits of the 19S complex (*Psmc4*, *Psmc2*, *Psmd11/Rpn6*, *Psmd8/Rpn12*, and *Psmd3*), 20S core particle (*Psma1*, and *Psmb3*), de-ubiquitinating gene (*Usp14*), ubiquitin-fusion protein (*Rps27a*), and the ubiquitin E3 ligase complexes, *Trim63* (MuRF1), *Socs3*, and F-box/WD repeat-containing protein 11 (*Fbxw11*). Although little is known about the substrates and functions of these E3 ligases in skeletal muscles, *Fbxw11* (also known as β-TrCP2) plays a role in targeting IkBα for ubiquitin-dependent degradation^29^. In addition, autophagy-related target genes (P62/*Sqstm1* and *Gabarapl1*), which are involved in protein degradation through the lysosomal/autophagy pathway, were upregulated in MG. In contrast, some of the atrogenes such as Atrogin-1/MAFbx (*Fbxo32*), Specific of Muscle Atrophy and Regulated by Transcription (SMART; *Foxo21*)^28^, splA/ryanodine receptor domain and SOCS box (*Spsb1*)^26^ were downregulated in MG compared with AG as shown in Fig. 3B.

P311/*Nrep,* which is involved in smooth muscle differentiation^30^ and reported to be downregulated in skeletal muscle after denervation^31^, was downregulated in MG compared with AG and GC (Fig. 3B). *Eif4ebp1*, which inhibits translation initiation factors, was upregulated in MG compared with AG and could indicate a reduction in translation in muscle^25^. A decrease in mRNAs for ECM proteins, Col1a1, was also observed in MG compared with AG. The transcription factor *Atf4*, which promotes the expression of oxidative stress-responsive genes and is induced during muscle atrophy^32^, was induced in MG. Thioredoxin-like protein and Metallothionein-1 (*Mt1*), which is upregulated during rodent sarcopenia^33^, were significantly upregulated in MG compared with AG and GC.

*Egr1* was significantly elevated in MG compared with GC, whose induction was significantly blocked in AG. However, the gene expression involved in ATP synthesis was not significantly changed among the three groups (Supplementary Table 2). Myogenin (*Myog*) is a muscle-specific transcriptional factor that is highly induced following muscle denervation^22^ and contributes to neurogenic muscle atrophy^34^. *Myog* was significantly increased in MG as compared with AG and GC. *Foxo1* and *Maf* were significantly decreased in MG as compared with AG and GC. Although the Kruppel-like factor 15 (*Klf15*) mediates the inhibition of mTOR in glucocorticoid-induced muscle atrophy through the induction upregulation of branched-chain amino acid transaminase 2 (*Bcat2*)^35^, the expression of *Klf15* and *Bcat2* was downregulated in MG compared with AG. The increase of *Klf15* was observed in AG compared with MG, despite no significant differences between GC and MG. This increased expression in AG compared with MG was also observed in Atrogin-1 (*Fbxo 32*), known as the target genes of *Klf15*.

In summary, the expressions of a set of atrogene were significantly changed in the soleus muscle under MG, and these altered expressions were blocked in that under AG. Atrogenes are known to be tightly and rapidly controlled, upregulated during atrophy, and returned to normal levels during recovery^36^. However, these results indicated that the expression of atrogenes was sustained in MG, although mice in MG were exposed to 1 *g* after returning to Earth. Conversely, some atrogenes such as *Klf15* were significantly increased in AG compared with MG, although the expression in MG was comparable to that in GC. It is possible that short-term exposure of AG mice to microgravity during the return phase might affect the gene expression. In addition, the expression of autophagy-related gene *Gabarapl1*is known to increase transiently both during atrophy and recovery following disuse muscle atrophy^37^. Moreover, activation of protein degradation such as ubiquitin–proteasome system was also observed in soleus muscle during early reloading stages (18 h.) after disuse muscle atrophy in rats^38^. It is still possible that the genes upregulated in MG have comprised recovery-related genes. Taken together, these results indicated that a set of atrogene expressions previously reported by ground experiments was changed under microgravity for 35 days, and that an artificial 1 *g* onboard environment could prevent the alteration of most atrogene expressions during spaceflight.

### Novel candidate genes associated with muscle atrophy were screened from gene expression profiles under spaceflight

**Figure 4:**
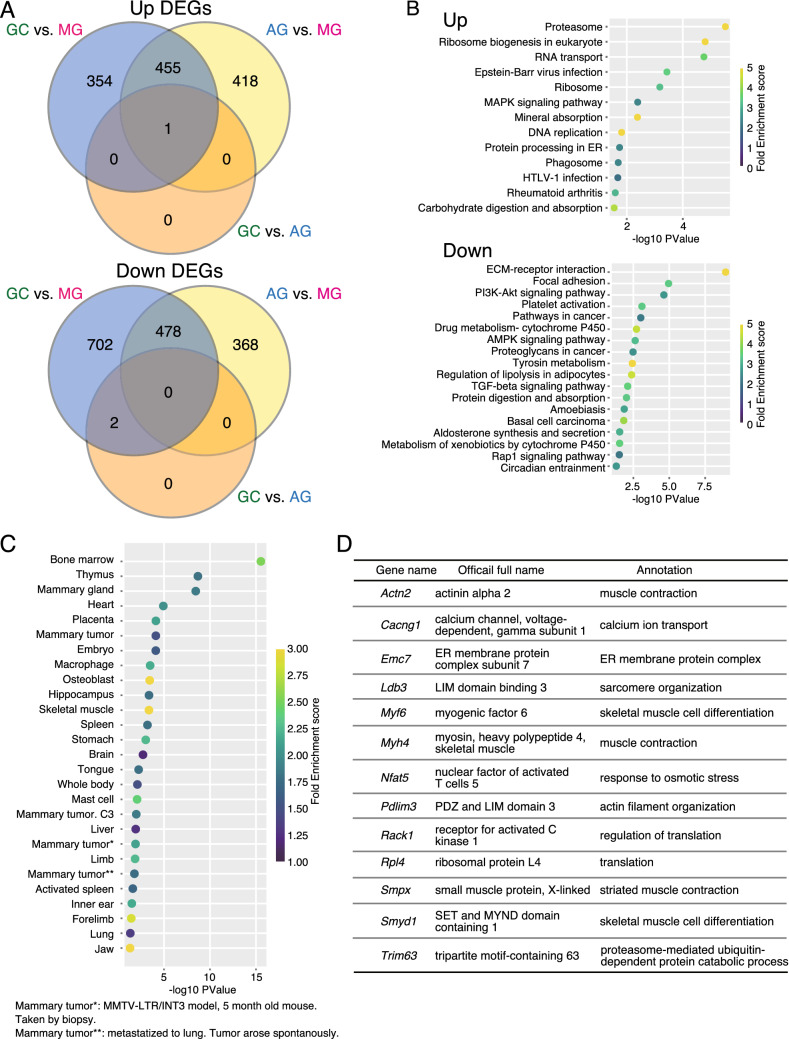
Pathway analysis of changed genes between MG and GC, and MG and AG, and identifying novel candidate genes involved in muscle atrophy. (**A**) Venn diagram showing the number of upregulated (upper) and downregulated (lower) DEGs in each comparison between MG, GC, and AG. (**B**) Pathway analysis of upregulated (upper) and downregulated (lower) DEGs in the overlapping region of MG compared with GC and AG. (**C**) Tissue expression profiles of 418 upregulated DEGs in MG compared with AG. (**D**) The gene list showing skeletal muscle-related genes from Fig. 4C.

Novel candidate genes associated with muscle atrophy were detected through a combination of comparative and in silico approach. Among the 1,992 genes differentially regulated in GC and MG (810 upregulated and 1,182 downregulated) and the 1,720 genes differentially regulated in AG and MG (874 upregulated and 846 downregulated), 455 genes were upregulated and 478 genes were downregulated, as shown in the overlapping region between the two comparisons, GC vs. MG and AG vs. MG in the Venn diagrams (Fig. 4A). To identify the functional gene clusters, we performed enrichment analysis for 455 upregulated genes and 478 downregulated genes in MG compared with GC and AG in the overlapping region between these two comparisons. The upregulated genes in MG were enriched in the pathways of the proteasome, ribosome biogenesis, RNA transport, MAPK signaling, mineral absorption, DNA replication, and lysosome (Fig. 4B). In contrast, downregulated genes in MG were enriched in the pathways of ECM-receptor interaction, focal adhesion, PI3K-Akt signaling, AMPK signaling, and TGF-β signaling. Consistent with this, downregulation of TGF-β1 was reported in the soleus muscle of mice on the ISS^39^. Also, decreased PI3K-Akt signaling was observed in the short-term spaceflight response in the gastrocnemius muscle in a previous study^40^. The upregulation of the proteasome pathway and the downregulation of the PI3K-Akt signaling pathway, which are involved in protein degradation and protein synthesis, respectively, were observed during muscle atrophy in MG.

Since AG was the new research condition under an artificial 1 *g* onboard environment, we screened the potential candidate genes involved in muscle atrophy under microgravity by focusing on genes differentially regulated between AG and MG. A total of 418 upregulated genes were significantly changed only specifically in the comparison between AG and MG, but not in that between GC and MG. The previous studies investigating the research on skeletal muscles by comparing spaceflight groups with ground control groups^6,12,13^ could not validate the effect of various factors during spaceflight, such as space radiation. The comparison between spaceflight and inflight control groups, instead of conventional control experiments, may provide new insights into understanding the mechanism of muscle atrophy during spaceflight. As the regulatory genes that induce muscular atrophy were found in upregulated genes in the previous reports^24^, we focused on the genes that were upregulated for identifying the novel molecule(s) that are involved in muscular atrophy under microgravity. Upregulated genes were distributed into the tissue expression profiles provided by the enrichment analysis (Fig. 4C). Thirteen skeletal muscle-related genes listed in Fig. 4D were significantly regulated in MG compared to AG in the soleus muscle. The functions of eight of the thirteen genes identified herein have been reported in muscle as follows: muscle development (*Actn2*), fiber type (*Myh4*), regeneration (*Nfat5*), dystrophy (*Ldb3*), and muscle atrophy (*Myf6, Trim63*, *Smyd1*, and *Rack1*). *Pdlim3* and *Smpx* were known not to affect the muscle when deficient *(Pdlim3*) or overexpressed in muscle (*Smpx*). *Actn2, Ldb3* and *Pdlim3* were known to be significantly upregulated in the cutaneous muscle of mice housed in the ISS for 91 days^41^, suggesting the potential role in diverse muscles during spaceflight. *Myh4* is one of the fast specific genes coding for MyHC type IIb. Upregulation of *Myh4* mRNA is consistent with the result that an increase in the ratio of type IIb fibers in MG was completely prevented in AG in the soleus muscle from MyHC staining, which is also reported in the soleus muscle of mice exposed to microgravity for 30 days on BION-M1^13^. NFAT5 has a role in both myoblast migration and differentiation during skeletal muscle myogenesis^42^, suggesting muscle regeneration in response to recovery after a return. Although *Myf6* is involved in differentiation as demonstrated by the temporal pattern of expression in embryos^43,44^, the role of *Myf6* in adult skeletal muscle is still elusive. *Myf6* was upregulated in both the soleus muscles of space-flown mice of the BION-M1 experiment^13^. Previous studies suggested that *Myf6* is involved in the gene expression reprogramming in denervated and regenerating muscle^45,46^. Therefore, *Myf6* could have a role in the response to muscle atrophy during spaceflight and/or recovery from microgravity. In addition, MuRF1 (*Trim63*) was increased and screened from this study. Although MuRF1 was upregulated in various ground-based models of muscle atrophy, muscle atrophy during spaceflight was not prevented in soleus muscle of MuRF1 KO mice in the previous study^21^.

On the other hand, the function of *Cacng1* remains unclear in skeletal muscle. *Cacng1* encodes γ1 subunit of the L-type Ca^2+^ channel, consisting of five subunits (α1S, α2, β, γ and δ) and is known as the dihydropyridine receptor (DHPR)^47,48^. DHPR is predominantly located in T-tables and interacts with the type 1 ryanodine sensitive Ca^2+^ release channel, RyR1, to release Ca^2+^ stored in the terminal cisternae of the sarcoplasmic reticulum for the initiation of contraction^49^. However, the biological significance of the DHPR is still unknown^50^. Therefore, we further validated whether *Cacng1* implicates muscle atrophy in vitro.

### Cacng1 induces a decrease in muscle fiber size in vitro and in vivo

**Figure 5:**
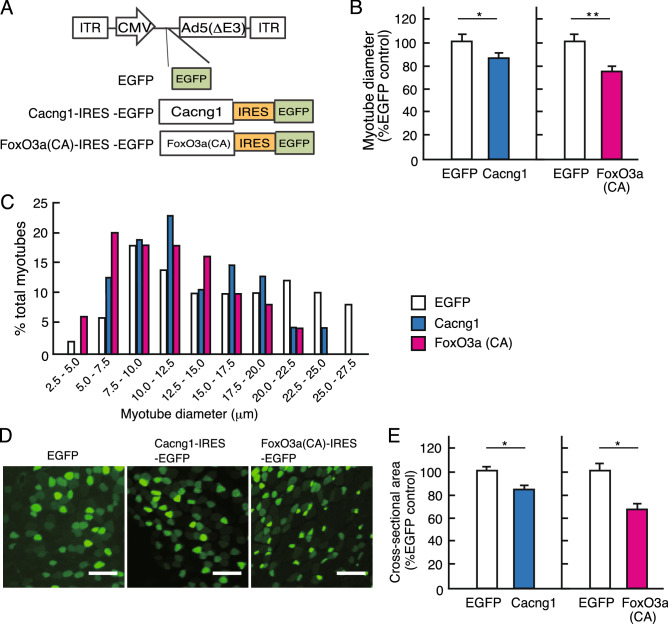
Functional validation of candidate genes using culture cell and neonatal mice muscle. (**A**) Construction of adenovirus vectors, only EGFP, Cacng1-IRES-EGFP, and FoxO3a (constitutive active; CA)-IRES-EGFP. (**B**) Quantification of the myotube diameters of C2C12 myotubes expressing EGFP, Cacng1-IRES-EGFP, and FoxO3a(CA)-IRES-EGFP. *P*-value from Student’s *t*-test is indicated as follows: *P < 0.05 and **P < 0.01. (**C**) Frequency distribution of myotube diameters in C2C12 myotubes expressing EGFP, Cacng1-IRES-EGFP, or FoxO3a(CA)-IRES-EGFP. (**D**) Representative cross-sectional areas of TA muscles expressing EGFP (Green) after the transfection of EGFP, Cacng1-IRES-EGFP, or FoxO3a(CA)-IRES-EGFP. Scale bars indicate 50 μm. (**E**) Bar diagrams showing the cross-sectional area of TA muscles expressing EGFP. *P*-value from Student’s *t*-test is indicated as follows: * *P* < 0.05. Three independent infections were performed in three mice.

Based on in silico analyses, we identified *Cacng1* as a novel candidate gene associated with muscle atrophy. Therefore, we further examined whether *Cacng1* induces muscle atrophy in vitro. C2C12 myotubes were infected with adenoviral vectors expressing *Cacng1* 4 days after differentiation when the myotubes are of constant size, and myoblast fusion no longer occurs^51^ (Fig. 5A). Morphological examination of the cells expressing *Cacng1* for 48 h. demonstrated a 15.2% reduction in mean myotube diameter and a leftward shift in myotube distribution compared with those expressing only EGFP control (Fig. 5B, C). Furthermore, when the myotubes were infected with a constitutively active mutant of FoxO3 (T32A, S252A, and S314A), FoxO3a (CA), a master regulator of muscle atrophy^52^, there was a 27.5% decrease in mean myotube diameter and a leftward shift in myotube distribution compared with those expressing only EGFP control (Fig. 5B, C). These findings demonstrate that the increased expression of *Cacng1* has a key role in the atrophy of myotubes in vitro.

To determine whether the induction of *Cacng1* expression influences fiber size in vivo, as it did in cultured myotubes, we conducted the gene transduction of *Cacng1* in neonatal mice’s skeletal muscle. Fiber sizes were measured 5 days after the *Cacng1*-expressing adenovirus vector was injected into neonatal mice’s TA muscle. Muscle fibers expressing FoxO3a (CA) shown in Fig. 5D were much smaller than those expressing only EGFP control. Mean CSA was markedly reduced in three individual experiments. *Cacng1* overexpression was reduced by 13.9% compared with the EGFP control, and FoxO3a overexpression was reduced by 32.2% compared with the EGFP control (Fig. 5D, E). These results suggested that *Cacng1* increased the distribution of thinner myotubes in the muscles, probably independent of the fiber type, since the increased *Cacng1* induces atrophy even in the fast-twitch TA muscle. Of note, the overexpression of Atrogin-1, a well-known atrogene involved in protein degradation, alone does not cause myotube or muscle atrophy^52^. Therefore, our findings suggest that *Cacng1* induces a pathway such as protein degradation and other atrophy-related changes that lead to pathways necessary to account for such marked fiber shrinkage.

In our study, we observed that *Cacng1* expression was significantly upregulated in mice exposed to microgravity compared with those exposed to an artificial 1 *g* onboard environment. We have shown that *Cacng1* induced muscle atrophy in both C2C12 cells and mouse neonatal skeletal muscles. These experiments in this study are the first to implicate the role of the excessive expression of γ1 subunit of the L-type calcium channel in the control of muscle fiber size.

Prolonged muscle inactivity during bed rest, hindlimb unloading, immobilization, microgravity, or denervation result in significant muscle atrophy^53–56^. It is also known that direct electrical stimulation preserved or restored almost normal properties in denervated muscle fiber likely triggered by action potentials or prompted by the contracted function^57,58^. A previous report showed that the downregulation of α1S subunit of DHPR in adult skeletal muscle by RNAi underwent massive atrophy, and that decreasing α1S induced redistribution of the neuronal nitric oxide synthase (nNOS), activation of FoxO3A, increased expression of atrogenes, and initiated autophagosome formation^50^. Given that γ1 subunit of DHPR is supposed to exhibit an endogenous ligand similar to Ca^2+^ antagonists and minimize Ca^2+^ entry and Ca^2+^ release from the previous study^49^, increased expression of γ1 subunit could induce muscle atrophy through similar mechanisms to the downregulation of α1S subunit. Further studies to reveal the mechanisms of increased expression of *Cacng1* to induce decreased fiber size are required to understand the contribution of *Cacng1* in muscle atrophy during spaceflight and other muscle atrophy on the ground.

In summary, an important asset of our experimental design is the comprehensive comparison of mice housed under microgravity or an artificial 1 *g* onboard environment, as an ideal control group to minimize any housing conditions-related bias, other than gravitational change during spaceflight. In previous studies, the antigravitational soleus muscle has often been studied in ground-experimental models of unloading to investigate the signaling pathways involved in skeletal muscle atrophy^59,60^ and microgravity effects on skeletal muscle^12,20^ due to its known susceptibility to unloading and disuse. However, further studies were required to understand the functional role of these genes by the effect of actual microgravity. Our results demonstrated that an artificial 1 *g* onboard environment in the ISS could prevent morphological changes and the alteration of gene expression under microgravity in mammals for the first time. Although CSA tend to decrease in AG compared with GC, the gene expression profile and expression of atrogene indicates that the muscle property of AG is similar to that of GC, consistent with the maintained muscle mass. These results suggested that loading of 1 *g* could be effective for maintaining muscle during spaceflight for at least 35 days. In particular, to the best of our knowledge, this is the first study in which soleus muscles collected from space-flown mice under microgravity and an artificial 1 *g* onboard environment were analyzed using RNAseq. These gene profiles could provide a novel and comprehensive overview of the effect of gravity loss on the global gene expression in space-flown mice’s soleus muscles. Therefore, we emphasized that the use of an artificial 1 *g* onboard environment can serve as a novel approach for identifying gene clusters that could be regulated by microgravity or other various environmental factors during spaceflight, such as space radiation. Furthermore, this study demonstrated that *Cacng1* is upregulated in atrophied soleus muscle during spaceflight, and the increased expression of *Cacng1* decreased the diameter of the myotube in vitro and in vivo. These results suggested that *Cacng1* might have a potential role in muscle atrophy during spaceflight.

However, our study has several limitations. First, it is necessary to consider the various possible conditions, such as the exposure against microgravity until splashdown and the post-landing effect of reloading due to the return of live animals. For example, given that AG mice were exposed to microgravity before splashdown for approximately 1.3 days because there was no centrifuge inside the rocket, it is possible that the short-term exposure of microgravity resulted in a significant increase in the gene expression of *Klf15* and its target gene Atrogin-1 (*Fbxo 32*) in the soleus muscle of AG mice compared with that of MG mice (Fig. 3). Apart from the short-term microgravity exposure of the AG mice before splashdown, the post-landing effect of reloading on the MG mice, which lasted for approximately 2 days, should also be considered in explaining the differences in the gene expression profiles of the soleus muscle in AG and MG mice. Second, although we demonstrated that the gene expression profiles between AG and GC are relatively similar in the soleus muscle, this study only reflects that spaceflight for 35 days has a larger effect on mice under microgravity than other events such as space radiation. Future studies may need to study different time points, such as longer periods using the MARS platform to elucidate the space radiation effects in mammals. Third, our study has not determined whether disuse or microgravity contributed mainly to muscle atrophy under spaceflight.

Taken together, we could gain a foothold in accelerating the estimation of muscle atrophy mechanisms by these gene profiles. Although the current study was made possible by state-of-the-art devices that implement an artificial 1 *g* onboard environment in the ISS, future studies of mammals will validate the effect of long-term habitation under gravitational forces weaker than 1 *g*, which is meant to simulate the gravity of the Moon and Mars, known as partial gravity. As experiment methods for space biology continue to develop, future studies may more conclusively identify the underlying causes and offer strategies to prevent muscle atrophy.

## Materials and methods

### Mice

All experiments were approved by the Institutional Animal Care and Use Committee of the University of Tsukuba (No. 16-048), JAXA (Protocol Number: 016-014B), Explora Biolabs (Study Number: EB15-010A), and NASA (Protocol Number: NAS-15-004-Y1) and were conducted according to the guidelines and applicable laws in Japan and the United States of America and the ARRIVE guidelines. Mice were maintained under specific pathogen-free conditions throughout the space experiment. The JAXA mission, MHU-1, was described previously^14^. In brief, C57BL/6 J male mice (Stock #000,664) were purchased from Jackson Laboratories (USA) for the space experiment and Charles River Laboratories (Japan) for the ground experiment. The SpaceX Falcon 9 rocket (SpX9) was launched on June 18, 2016 (EDT) from the Kennedy Space Center (KSC). Inside the ISS, 12 mice (8 weeks old) were divided into two groups: artificial 1 *g* (AG) and microgravity (μ*g*; MG) and housed for 34.6 days in Habitat Cage Units (HCUs) under μ*g* conditions and for 34.1 days and under 1 *g* during the approximate 37-day berthing period. They were housed for 35 days in HCUs and then returned to Earth in specific pathogen-free conditions. All mice splashed down in the Pacific Ocean near the West Coast on August 26, 2016 and were then transported to the Explore Biolabs laboratory (San Diego) for behavioral observation and dissection 2 days later. The GC experiment that replicated the housing conditions of the flight experiment was conducted at the JAXA Tsukuba Space Center (TKSC) in Japan from August 27 to November 3, 2016. All mice were euthanized by inhalation of 2% isoflurane, and then dissected to collect tissue samples.

### Histological staining of HE and immunostaining of the different muscle fiber types

The soleus muscles were immediately frozen in isopentane cooled with liquid nitrogen. Frozen tissues (8 μm in thickness) were mounted on glass slides and subjected to hematoxylin–eosin staining. Immunohistochemical analysis of the different muscle fiber types was performed using fiber type-specific antibodies (Type I; BA-D5, Type IIa; SC-71, Type IIb; BF-F8, the Developmental Studies Hybridoma Bank (DSHB)). The tissue sections were treated with a Mouse on Mouse (M.O.M.) fluorescein kit (Vector Laboratories) to block mouse IgG background. Immunodetection was performed using Alexa Fluor-conjugated secondary antibodies (Alexa Fluor 350 conjugated anti-mouse IgG2b, Alexa Fluor 488 conjugated anti-mouse IgG1, Alexa Fluor 555 conjugated anti-mouse IgM, Thermo Fisher). Images were captured and analyzed using a BIOREVO BZ-X800 microscope system (Keyence, Osaka, Japan). The number of immunoreactive muscle fibers and CSAs were determined using the hybrid cell count software by Keyence.

### RNA sequencing

RNAseq was employed for transcriptome analysis. RNA was extracted from the soleus muscles of 3 mice in each group: AG, MG, and GC. Briefly, total RNA was isolated using the TRIZOL reagent (Thermo Fisher Scientific). RNA quality was controlled using an RNA 6000 Pico kit (Agilent, Santa Clara, CA, USA) from 100 frozen sections (8 μm in thickness) of the soleus muscle. A total amount of 50 ng total RNA was used for RNAseq library preparation using the NEBNext rRNA Depletion Kit and NEBNext Ultra Directional RNA Library Prep Kit (New England Biolabs, Ipswich, MA, USA); 2 × 36 base paired-end sequencing was performed using NextSeq500 (Illumina, San Diego, CA) by Tsukuba i-Laboratory LLP (Tsukuba, Ibaraki, Japan). Sequence reads were mapped into the mouse genome (mm10) and quantified for annotated genes using the CLC Genomics Workbench (Version 11.0.2; Qiagen, Redwood City, CA, USA). The expression levels were calculated in transcripts per million (TPM)^61^. We excluded genes with less than 10 read counts in any sample. After filtering, the differential gene expression was calculated between conditions (GC vs. MG, AG vs. MG, GC vs. AG) with the FDR-corrected *P* < 0.05 and a 1.5-fold change cutoff using the empirical analysis of DGE tool (EdgeR) on the CLC Genomics Workbench software.

### Enrichment analysis process

DAVID Bioinformatics Resources 6.8^62^ was used for pathway analysis and for enrichment analysis of the tissue specificity database (UniProt; UP-Tissue) with *P*-value < 0.05.

### Generation of the adenoviral vector

Three recombinant adenoviruses encoding the EGFP alone, mouse Cacng1 or FoxO3a (CA), a constitutively active (CA) form of mouse FoxO3a, fused with the IRES-EGFP cassette expressing the green fluorescent protein were generated by in vitro recombination techniques. Briefly, the IRES-EGFP cDNA was amplified from the pIRES2-EGFP vector (BD Biosciences Clontech) using the following primers: forward, 5′-CCCACTCGAGGCCCCTCTCCCTCC-3′; reverse, 5′-GGGTGATATCTTACTTGTACAGCTC-3′. *Xho* I and *Eco*RV restriction sites are underlined. The amplified fragment was inserted at the *Xho* I-*Eco*RV of the pENTR3C entry vector to generate the pENTR3C-IRES-EGFP entry vector. *Cacng1* cDNA was cloned by RT–PCR from mouse soleus muscle using the following primers: forward, 5′-CCCAGGATCCGCCACCATGTCACAGACC-3′; reverse, 5′-GGGTCTCGAGCTAGTGCTCTGGCTC-3′. *Bam*HI and *Xho* I restriction sites are underlined. The amplified fragment was inserted at the *Bam*HI and *Xho* I sites of the pENTR3C-IRES-EGFP entry vector to generate the pENTR3C-Cacng1-IRES-EGFP vector. pcDNA3-Flag-mouse FoxO3a was a generous gift of Prof. A. Fukamizu (University of Tsukuba). The expression vectors for FoxO3a(CA) mutated at the three AKT phosphorylation sites (T32A, S252A, S314A) were constructed by introducing the mutations by PCR-based site-directed mutagenesis. Flag-FoxO3a-CA was cloned by RT–PCR from mouse soleus muscle using the following primers: forward, 5′-CCCCGAATCCGCCACCATGGATTATAAGGAC-3′; reverse, 5′-GAGGGGCAAACAACAGATGG-3′. *Eco*RI restriction sites are underlined. The amplified fragment was inserted at the *Eco*RI sites of the pENTR3C-IRES-EGFP entry vector to generate pENTR3C-Flag-FoxO3a(CA)-IRES-EGFP vector. Then, these inserts were transferred into the pAd/CMV/V5-DEST (Thermo Fisher Scientific) adenoviral expression vector using the ViraPower Adenoviral Gateway Expression Kit (Life Technologies, Carlsbad, CA) according to the manufacturer’s instructions. All constructs were sequenced to confirm their identity. The recombinant plasmids obtained, pAd-EGFP, pAd-Cacng1-IRES-EGFP, and pAd-Flag-FoxO3a(CA)-IRES-EGFP, were linearized with *Pac* I (New England Biolabs Japan Inc., Tokyo, Japan) restriction enzyme, and ethanol-precipitated. They were then used for the transfection of 293A cells with Lipofectamine 2000 (Invitrogen, Carlsbad, CA, USA) in Opti-MEM medium (Invitrogen) on collagen type I-coated dishes (Thermo Fisher Scientific) to produce the recombinant adenoviral vectors. 4–6 h. after incubation at 37 °C, the medium with the transfection mix was removed, and the growth medium was added. The transfected cells were monitored for EGFP expression and collected 7–14 days after transfection by scraping cells off the dishes and pelleting them along with any floating cells in the culture. After three cycles of freezing in liquid nitrogen and rapid thawing at 37 °C, the viral lysate was used to infect the new cells. One to two days later, the viruses were harvested as described above. This process was repeated 5 times; then, the viruses were purified by cesium chloride (CsCl) gradient ultracentrifugation followed by dialysis with three changes of buffer containing sodium chloride and magnesium chloride to remove CsCl. Viral titers were determined by a plaque-forming assay using the Adeno-X Rapid Titer Kit (Takara bio.) and 293 cells according to the manufacturer’s instructions.

### Cell culture, adenoviral infection, and myotube analysis

The C2C12 myoblast cell line (ATCC; CRL-1772) was cultured in high-glucose Dulbecco’s modified Eagle’s Medium (DMEM) supplemented with 10% fetal bovine serum until the cells reached confluence. The medium was then replaced with DMEM 2% horse serum (HS; Thermo Fisher Scientific), the differentiation medium, and incubated for 4 days to induce myotube formation before proceeding with the experiments. For infection, the myotubes were incubated with adenovirus at a multiplicity of infection (MOI) of 250, 125, and 62.5 in the differentiation medium for 18 h., and then the medium was replaced with the differentiation medium without adenovirus. The infection efficiency was typically greater than 90%. Two days after infection, the cells were fixed in 4% paraformaldehyde/PBS for 10 min. and permeabilized in 0.1% Triton X-100/PBS for 10 min. The fixed cells were blocked for 1 h. in 2% BSA/PBS and incubated overnight at 4 °C with anti-myosin heavy chain (MHC) monoclonal antibody (1:100; MF20, DSHB). The antigens were visualized using appropriate secondary antibodies conjugated with Alexa Fluor 546 (1:200; Thermo Fisher Scientific), and the nuclei were labeled with Hoechst 33,342 (Molecular Probes). The cells were mounted with 20 mM Tris-HCl (pH 8.0), 90% glycerol. All images were captured by using a fluorescence microscope (BIOREVO BZ-9000, Keyence). The myotube diameter was quantified by measuring a total of > 100 tube diameters from ten random fields at 200 × magnification using ImageJ software (NIH, Frederick, MD, USA) as described^51^. All data are expressed as the mean ± SEM.

### Adenoviral vector administration and fiber size measurements

Adenoviral vector administration and fiber size measurements were performed as previously described^63^. Briefly, 2- to 4-day-old ICR mice pups (Japan SLC Inc.) were anesthetized via hypothermia by placement on ice-cooled aluminum foil for a few minutes. Infectious particles (1–2 × 10^7^) of Ad5 cytomegalovirus containing mouse Cacng1-IRES-EGFP, FoxO3a(CA)-IRES-EGFP, or EGFP cDNA diluted in a final volume of 10 μl in PBS were injected percutaneously into each skeletal muscle of the corresponding mice using an insulin syringe. The pups were reintroduced to the mother and kept in quarantine for 5 days. All pups survived after infection.

### Statistics

All data are presented as the mean ± SEM. The statistical significance between GC, AG, and MG (Fig. 1B, E, F) were determined by Tukey’s test. Results with *P* < 0.05 were considered significant. The comparison between EGFP and Cacng1, and EGFP and FoxO3a(CA) were determined using Student’s *t*-test, and results with *P* < 0.05 were considered significant (Fig. 5B, E).

## Supplementary Information

Supplementary Information 1.

Supplementary Information 2.

Supplementary Information 3.

## Data Availability

All data that support the findings of this study are available from the corresponding author upon reasonable request. RNAseq data are deposited in the DDBJ database (The DNA Databank of Japan, https://www.ddbj.nig.ac.jp/) (accession number DRA010983).
