# Supplementary material for: Transcriptome analysis of gravitational effects on mouse skeletal muscles under microgravity and artificial 1 g onboard environment
Source: Sci Rep. 2021 Apr 28;11:9168. doi: 10.1038/s41598-021-88392-4 (PMC8080648; doi:10.1038/s41598-021-88392-4)
Supplement: Supplementary file 1 — Supplementary Information 1. [file 41598_2021_88392_MOESM1_ESM.pdf]

Transcriptome analysis of gravitational effects on mouse skeletal muscles under microgravity and artificial 1 *g* onboard environment

Risa Okada[1,2], Shin-ichiro Fujita[3,4], Riku Suzuki[5,6], Takuto Hayashi[3,5], Hirona Tsubouchi[5], Chihiro Kato[5,7], Shunya Sadaki[5], Maho Kanai[5,6], Sayaka Fuseya[3,5], Yuri Inoue[3,5], Hyojung Jeon[5], Michito Hamada[5], Akihiro Kuno[5,6], Akiko Ishii[8], Akira Tamaoka[8], Jun Tanihata[9], Naoki Ito[10], Dai Shiba[1,2], Masaki Shirakawa[1,2], Masafumi Muratani[1,4], Takashi Kudo[1,5]*, Satoru Takahashi[1,5]*

[1]Mouse Epigenetics Project, ISS/Kibo experiment, Japan Aerospace Exploration Agency (JAXA), Ibaraki 305-8505, Japan.

[2]JEM Utilization Center, Human Spaceflight Technology Directorate, JAXA, Ibaraki 305-8505, Japan.

[3]Doctoral Program in Biomedical Sciences, Graduate School of Comprehensive Human Sciences, University of Tsukuba, Ibaraki 305-8575, Japan.

[4]Department of Genome Biology, Faculty of Medicine, University of Tsukuba, Ibaraki 305-8575, Japan.

[5]Laboratory Animal Resource Center in Transborder Medical Research Center, and Department of Anatomy and Embryology, Faculty of Medicine, University of Tsukuba, Ibaraki 305-8575, Japan.

[6]Ph.D. Program in Human Biology, School of Integrative and Global Majors, University of Tsukuba, Ibaraki 305-8575, Japan.

[7]Master's Program in Medical Sciences, Graduate School of Comprehensive Human Sciences, University of Tsukuba, Ibaraki 305-8575, Japan.

[8]Department of Neurology, Faculty of Medicine, University of Tsukuba, Ibaraki 305-8575, Japan.

[9]Department of Cell Physiology, The Jikei University School of Medicine, Tokyo, 105-8461, Japan.

[10]Laboratory of Molecular Life Science, Institute of Biomedical Research and Innovation, Foundation for Biomedical Research and Innovation at Kobe (FBRI), Kobe 650-0047, Japan.

*Corresponding authors: t-kudo@md.tsukuba.ac.jp; satoruta@md.tsukuba.ac.jp.

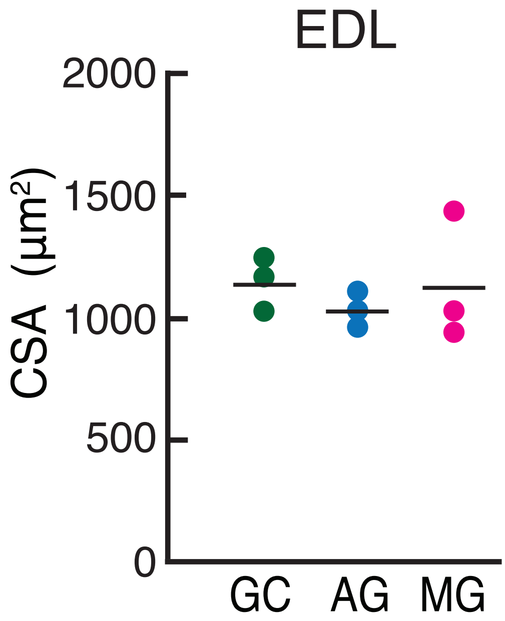

Supplementary Figure 1.

Quantification of cross-sectional areas (CSAs) of EDL muscles in the GC, AG, and MG.

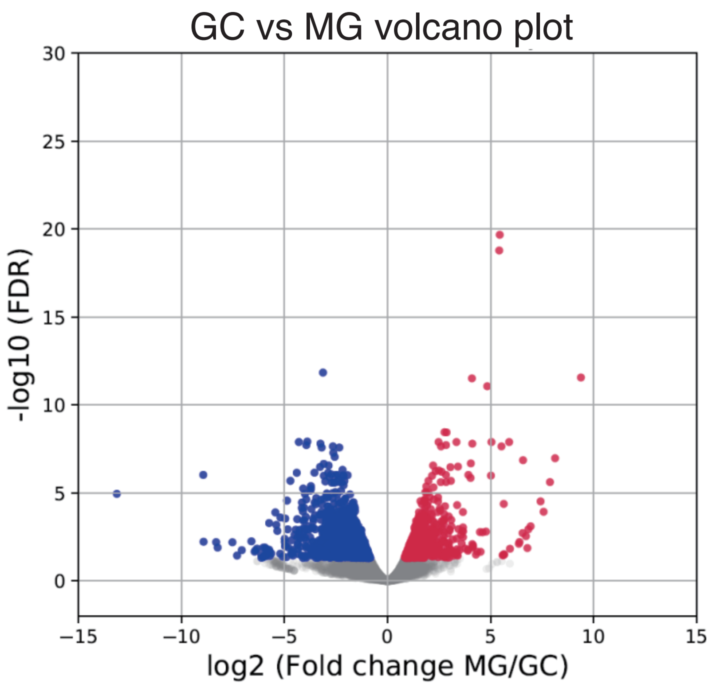

Supplementary Figure 2.

Volcano plots showing differentially regulated genes in the soleus muscle of GC and MG.

Supplementary Table 1

Gene lists of differentially expressed genes in the soleus muscle among MG, AG and GC.

Supplementary Table 2

Gene lists of differentially expressed atrogenes in the soleus muscle among MG, AG and GC.
